# A potential molecular model for studying apoptosis enhanced by the interaction of BCL-G with JAB1 in swine

**DOI:** 10.18632/oncotarget.11230

**Published:** 2016-08-11

**Authors:** Pengfei Jiang, Xingye Wang, Xiaolin Chen, Yaping Wang, Zhanzhan Kang, Jingna Wang, Deli Zhang

**Affiliations:** ^1^ Department of Microbiology & Immunology, School of Basic Medical Sciences, Wenzhou Medical University, Wenzhou, Zhejiang, P. R. China; ^2^ College of Veterinary Medicine, Northwest A&F University, Yangling, Shaanxi, P. R. China

**Keywords:** porcine BCL-G, spatial expression, subcellular localization, interaction with porcine JAB1, enhancement to apoptosis, Autophagy

## Abstract

BCL-G, an apoptotic factor in Bcl-2 family, is involved in several kinds of diseases by interacting with several proteins. Although many studies on mouse and human BCL-G have been reported, porcine BCL-G (pBCL-G) has been little investigated. In this study, our results showed that pBCL-G was universally expressed in porcine tissues. The BH2 domain affected the subcellular distribution of pBCL-G protein. pBCL-G could interact with porcine JAB1 (pJAB1), by which its subcellular distribution was affected. pBCL-G promoted staurosporine-induced apoptosis that was significantly enhanced by interaction of pBCL-G with pJAB1. The apoptosis at least partially depended on the activated caspase-8, -9 and -3. Owing to the close phylogenetic distance between pigs and humans and their many physiological similarities, our findings may provide a potential molecular model to study human BCL-G and also may have implications in the treatment of diseases relevant with BCL-G.

## INTRODUCTION

Apoptosis is a defense mechanism that can eliminate cells ruined by kinds of factors [1]. The intrinsic pathway is one of the many demonstrated pathways involved in apoptosis [2]. Proteins in Bcl-2 family, featured with the typical Bcl-2 homology (BH) domains, consist of pro- and anti-apoptotic factors, by which the intrinsic apoptotic pathway is regulated [2]. BCL-G, a member from Bcl-2 family and originally identified as a pro-apoptotic protein in humans [3], has been implicated in several diseases by interacting with many proteins [3–8]. However, in a recent study, mouse BCL-G was reported not to be a pro-apoptotic protein [9]. Taken together, these data indicate that BCL-G protein has different functions in different species.

Staurosporine (STS), an apoptosis inducer [10–16], can activate the apoptotic pathway mediated by mitochondrion [17]. A large numbers of proteins can regulate the STS-induced apoptosis [16, 18–20]. In our recent study, porcine JAB1 (pJAB1) was found to enhance STS-induced apoptosis [11].

Although many studies on mouse and human BCL-G have been reported, porcine BCL-G (pBCL-G) has been little investigated. It has been reported that human JAB1 could bind to BCL-G_S_ and that N-terminal 67 amino acids of BCL-G_S_ was both sufficient and necessary for the interaction [4]. BCL-G_S_ is one protein encoded by BCL-G gene. By alternative mRNA splicing, BCL-G gene could encode another protein, BCL-G_L_, whose first 226 amino acids are identical to BCL-G_S_ [3]. According to our previous study, pBCL-G is similar to human BCL-G_L_ with 71% identity [21] and pJAB1 is absolutely identical with human JAB1 [11]. Does pBCL-G interact with pJAB1? Is pBCL-G involved in STS-induced apoptosis? In the present study, we clarify these questions by exploring the interaction of pBCL-G with pJAB1 and its role in STS-induced apoptosis.

Because pigs have many physiological similarities with humans [22–25], they are optimal animals for scientific research and have been widely used as medical models to study human diseases [26–29]. Our results may provide not only useful information on the pBCL-G gene but also a potential molecular model to study human diseases correlated with BCL-G.

## RESULTS

### The expression pattern of pBCL-G at transcript level

**Figure 1:**
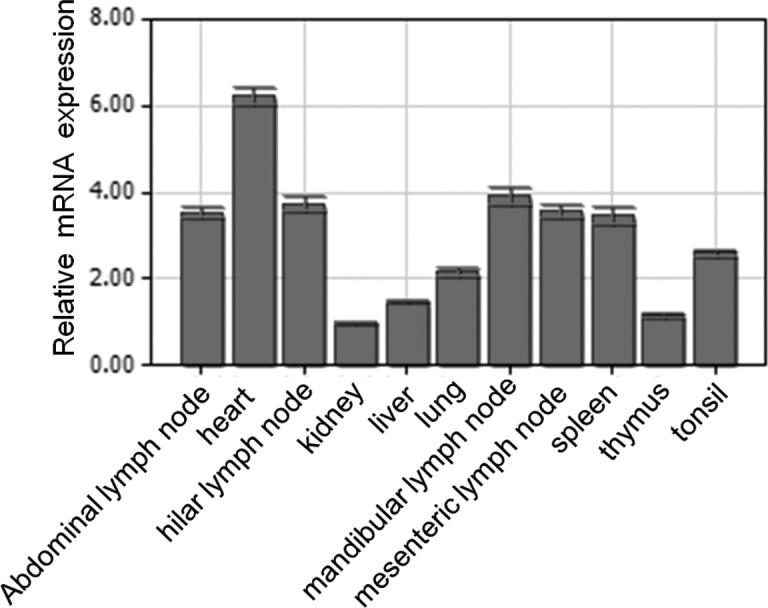
Spatial expression analysis of pBCL-G mRNA by real-time PCR Mean mRNA levels in eleven tissues were analyzed. The values were normalized to endogenous β-actin expression. All experiments were performed in triplicate and data are expressed as means ± SD (*n* = 3). Error bars represent standard deviations of replicate data points.

Quantitative real-time PCR was used to analyze the spatial expression of the pBCL-G mRNA in different swine tissues. As shown in Figure 1, pBCL-G was widely expressed in the detected organs and tissues. Among these samples, heart exhibited the highest expression level, followed by mandibular lymph node, abdominal lymph node, hilar lymph node, mesenteric lymph node, spleen, lung, liver, and thymus. The lowest expression level was shown in kidney. There is a more than 6-fold difference between heart and kidney.

### The BH2 domain affects the subcellular distribution of the pBCL-G protein

**Figure 2:**
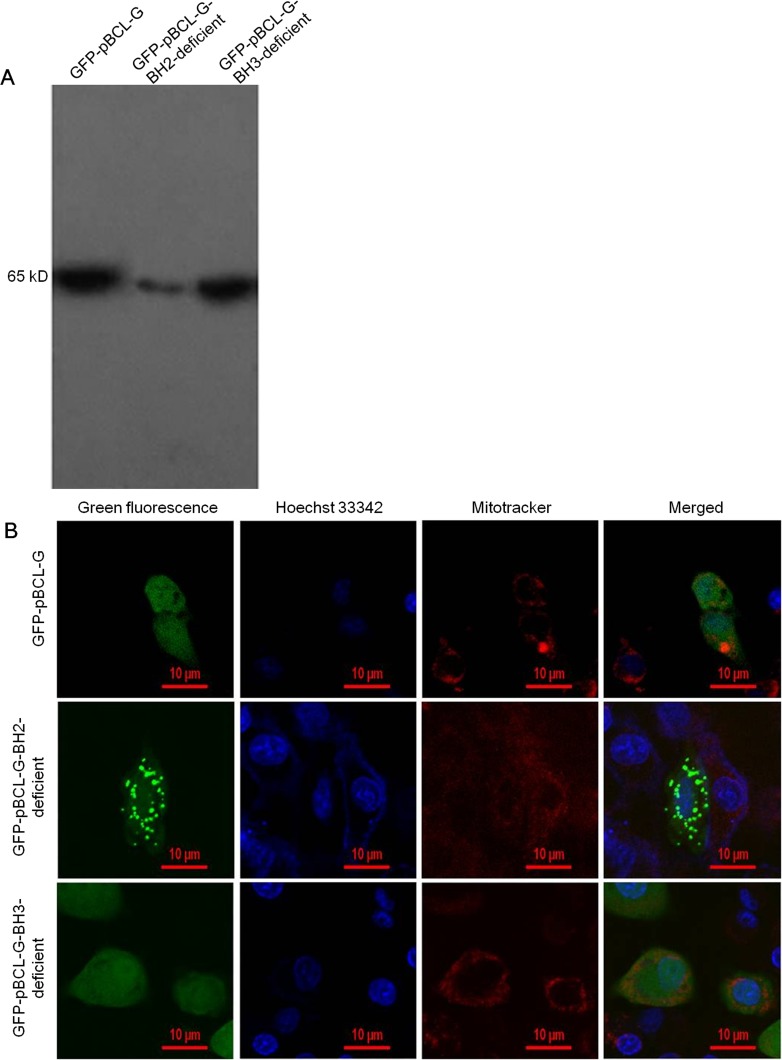
Detection of the expression and subcellular localization of GFP-fused complete and two mutant pBCL-G proteins **A.** Western blot analysis of GFP-fused complete and two mutant pBCL-G proteins expression using anti-GFP tag antibody. **B.** Confocal microscopy images of SUVEC cells expressing GFP-fused complete and two mutant pBCL-G proteins. All of the cell lines were stained by Hoechst 33342 and MitoTracker^®^ Red CMXRos. Merged images showed co-localization of the GFP-pBCL-G-BH2-deficient protein with the mitochondrion. Bar = 10 μm for all the figures.

To explore the effect of pivotal domains on the subcellular distribution of pBCL-G, two mutant genes expressing pBCL-G proteins lacking the BH2 or BH3 domain respectively were created and cloned into pEGFP-C1 vector to construct pEGFP-pBCL-G-BH2-deficient or pEGFP-pBCL-G-BH3-deficient vector. The pBCL-G gene was cloned into the pEGFP-C1 vector to construct the pEGFP-pBCL-G vector as previously described [21]. Then, the two mutant expressing vectors were transfected into Swine umbilical vein endothelial cells (SUVEC), respectively. The pEGFP-pBCL-G vector was used as a control. Western blot analysis was performed to detect the expression of these proteins (Figure 2A). The subcellular localization of these two mutant pBCL-G proteins was detected by confocal microscopy analysis. As shown in Figure 2B, the mutant pBCL-G protein lacking BH2 domain was distributed in a punctate cytosolic pattern whereas the mutant pBCL-G protein lacking BH3 domain and the complete pBCL-G protein were both distributed throughout cells.

### Validation of cell clone stably expressing the GFP-pBCL-G protein

**Figure 3:**
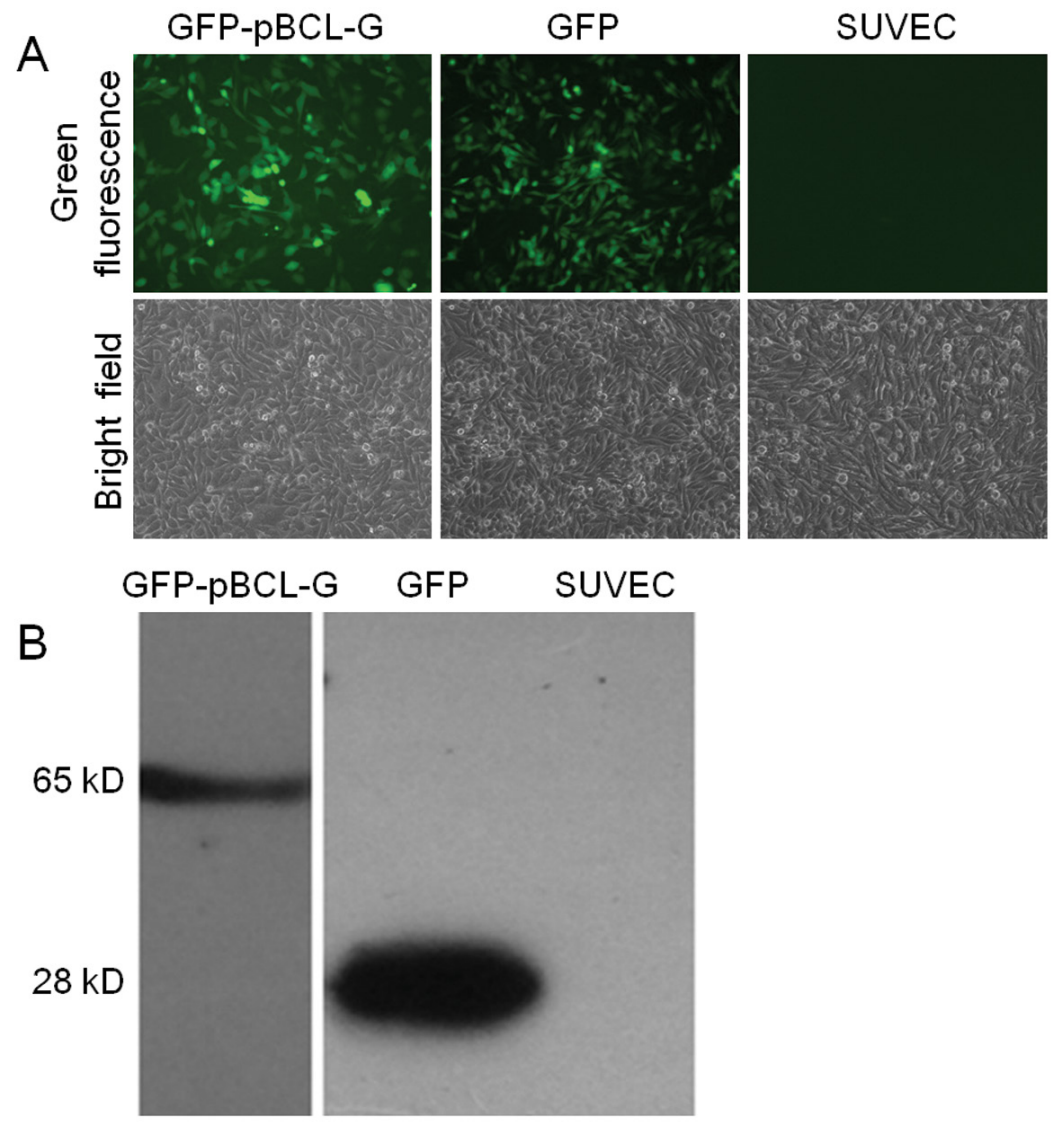
Validation of SUVEC cell clones stably expressing GFP-pBCL-G or GFP proteins (A) Detection of GFP-pBCL-G or GFP proteins stably expressed in SUVEC cells by inverted fluorescence microscopy. (B) Detection of GFP-pBCL-G or GFP proteins stably expressed in SUVEC cells by western blot analysis.

To study the function of pBCL-G conveniently, the pEGFP-pBCL-G vector was transfected into SUVEC cells, and then the transiently transfected cells were selected using G418. One cell clone was obtained after 2 weeks of selection. Initially, the GFP-pBCL-G protein in the selected cell clone was detected by Inverted fluorescence microscopy (Figure 3A). Then, a protein corresponding to the size of the GFP-BCL-G fusion protein was identified by western blot analysis, confirming the expression of GFP-pBCL-G in this cell clone (Figure 3B). Another cell clone that could stably express the GFP protein was generated as a control using the similar method.

### pBCL-G can interact with pJAB1

**Figure 4:**
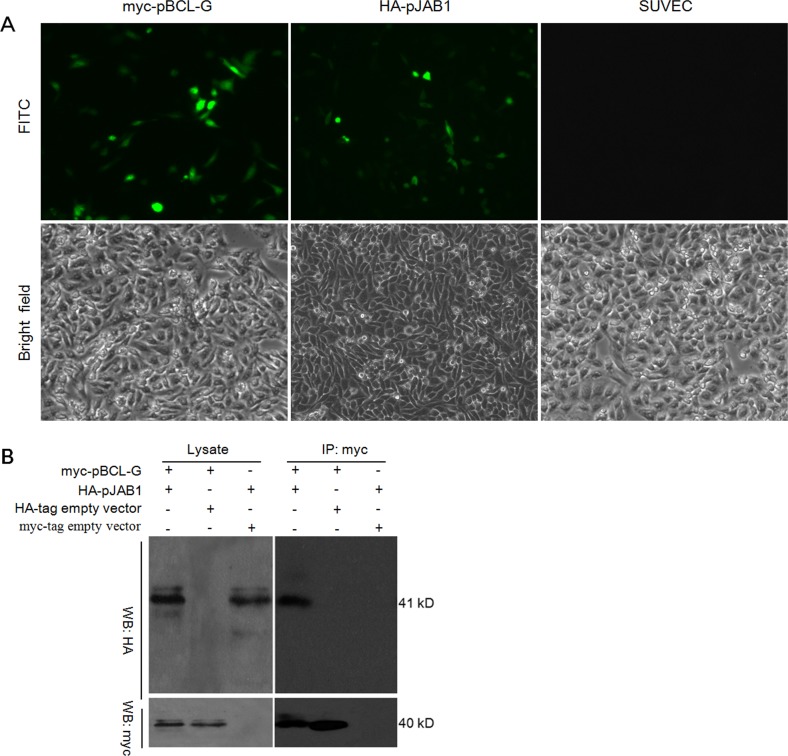
Detection of the interaction between pBCL-G and pJAB1 **A.** Detection of myc-pBCL-G and HA-pJAB1 proteins expression by indirect immunofluorescence. **B.** Detection of the interaction between pBCL-G and pJAB1 by co-immunoprecipitation.

To explore the interaction of pBCL-G with pJAB1, we performed a co-immunoprecipitation assay. Both myc-tagged pBCL-G (myc-pBCL-G) and HA-tagged pJAB1 (HA-pJAB1) were over-expressed in SUVEC cells. The indirect immuno-fluorescence assay was performed to detect indicated proteins, with western blot for confirmation. Then, co-immunoprecipitation was carried out. As shown in Figure 4A, fluorescein isothiocyanate (FITC) fluorescence signal was detected in cells that could express myc-pBCL-G and HA-pJAB1 proteins, whereas the signal was not detected in normal cells. By western blot analysis, HA-pJAB1 could be detected in the immunoprecipitated product when myc-pBCL-G and HA-pJAB1 were concurrently over-expressed in cells. However, HA-pJAB1 could not be detected in the immunoprecipitated product when myc-pBCL-G and HA-tag were concurrently over-expressed in cells and the same result was obtained when myc-tag and HA-pJAB1 were concurrently over-expressed in cells (Figure 4B). These results indicated that pBCL-G could interact with pJAB1.

### pJAB1 affects the subcellular localization of pBCL-G

**Figure 5:**
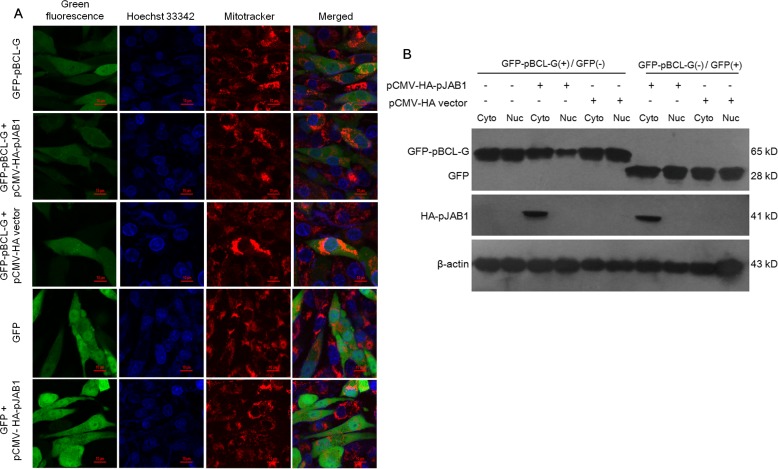
pJAB1 affects the subcellular localization of pBCL-G protein **A.** Subcellular localization of stably expressed GFP-pBCL-G proteins was detected by confocal microscopy before and after transfection with pCMV-HA-pJAB1 or pCMV-HA vector. All of the cell lines were stained by Hoechst 33342 and MitoTracker^®^ Red CMXRos. Merged images showed changes of the subcellular localization of GFP-pBCL-G protein. Bar = 10 μm for all the figures. **B.** Nuclear and cytoplasmic fractions derived from SUVEC cells stably expressing the GFP-pBCL-G protein and transfected with pCMV-HA-pJAB1 or pCMV-HA empty vector were analyzed by western blot. The change of the subcellular localization of GFP-pBCL-G protein was shown by the expression level of this protein in the cytoplasmic and nuclear fractions when cells were transfected with the pCMV-HA-pJAB1 vector. GFP-pBCL-G(+) and GFP(+) meant cells stably expressing GFP-pBCL-G and GFP proteins, respectively. The GFP-pBCL-G, GFP and HA-pJAB1proteins were detected using anti-GFP tag and anti-HA tag antibodies, respectively. The data shown are representative of three independent experiments.

The finding that pBCL-G interacts with pJAB1 *in vivo* leads us to speculate that pJAB1 may affect the subcellular localization of pBCL-G. To assess this hypothesis, pCMV-HA-pJAB1 or pCMV-HA empty vector was respectively transfected into SUVEC cells that could stably express the GFP-pBCL-G protein. As shown in Figure 5A, the GFP-pBCL-G protein was normally distributed in both cytoplasm and nucleus whereas the corresponding protein was distributed mainly in the cytoplasm when pCMV-HA-pJAB1 vector was transfected into cells, which was consistent with the subcellular distribution of pJAB1 [11]. When cells were transfected with pCMV-HA vector, the distribution of GFP-pBCL-G protein was same as that in cells without transfection. As a control, SUVEC cells that could stably express the GFP protein were also transfected with pCMV-HA-pJAB1 vector. However, the subcellular distribution of GFP protein was not affected by the transfection.

To confirm the above results, pCMV-HA-pJAB1 or pCMV-HA empty vector was respectively transfected into SUVEC cells that could stably express the GFP-pBCL-G protein. Then, the nuclear and cytoplasmic fractions derived from these cells were analyzed by western blot. The results showed that the amount of GFP-pBCL-G protein in cytoplasmic fraction was significantly more than that in nuclear fraction when pCMV-HA-pJAB1 vector was transfected into cells that could stably express GFP-pBCL-G protein, whereas the amount of corresponding protein in both cytoplasmic and nuclear fractions from cells transfected with pCMV-HA vector had no difference with that of the untransfected cells. In SUVEC cells that could stably express GFP protein, transfection of pCMV-HA-pJAB1 or pCMV-HA vector had no effect on GFP protein. In addition, the expression of HA-pJAB1 was only detected in the cytoplasmic fraction. These results were consistent with the results obtained by confocal microscopy (Figure 5B).

### pBCL-G promotes STS-induced apoptosis

**Figure 6:**
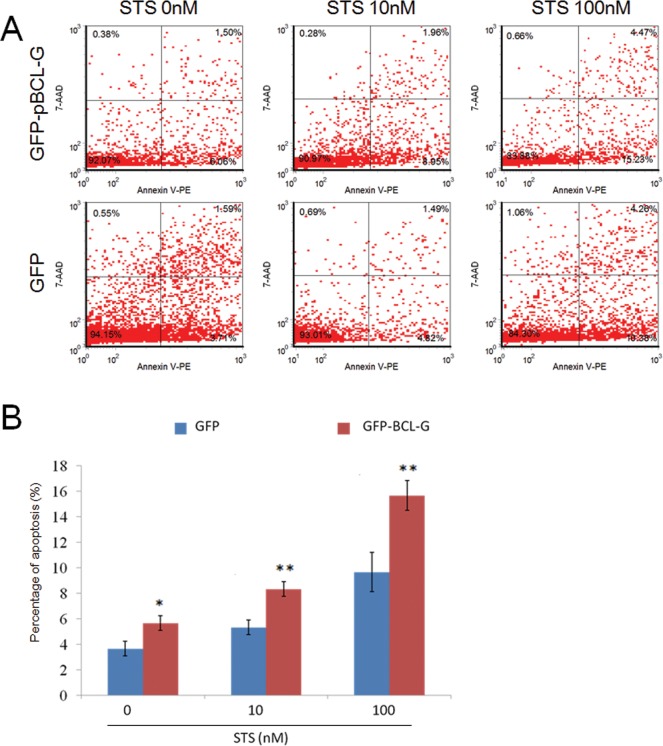
Quantification of apoptosis enhanced by pBCL-G **A.** Representative FCM diagrams of SUVEC cells transiently expressing GFP-pBCL-G or GFP proteins treated with the corresponding STS doses for 18 hours. **B.** Quantification of annexin V-positive cells. All experiments were performed in triplicate and data are expressed as means ± SD (*n* = 3). Error bars represent standard deviations of replicate data points. ^*^p < 0.05, ^**^p < 0.01 *versus* the control group (Cells expressing GFP proteins).

To clarify the function of pBCL-G in STS-induced apoptosis, flow cytometry (FCM) combined with annexin V-PE/7-AAD staining was applied to distinguish necrotic cell death from apoptosis. As shown in Figure 6A, SUVEC cells that could transiently express GFP-pBCL-G protein were incubated with annexin V-PE to detect the phosphatidylserine externalization of the cell membrane as the early apoptosis marker after cells were incubated with 0-100 nM STS for 18 hours. In the FCM diagrams, the lower right quadrant meant early apoptosis, whereas the upper right quadrant meant late apoptosis. SUVEC cells transiently expressing GFP with the same treatment were used as a control. As shown in Figure 6B, compared with cells expressing GFP protein, there was a significant increase in early apoptosis when cells expressing GFP-pBCL-G protein were treated with indicated doses of STS.

### The interaction of pBCL-G with pJAB1 significantly enhances STS-induced apoptosis

**Figure 7:**
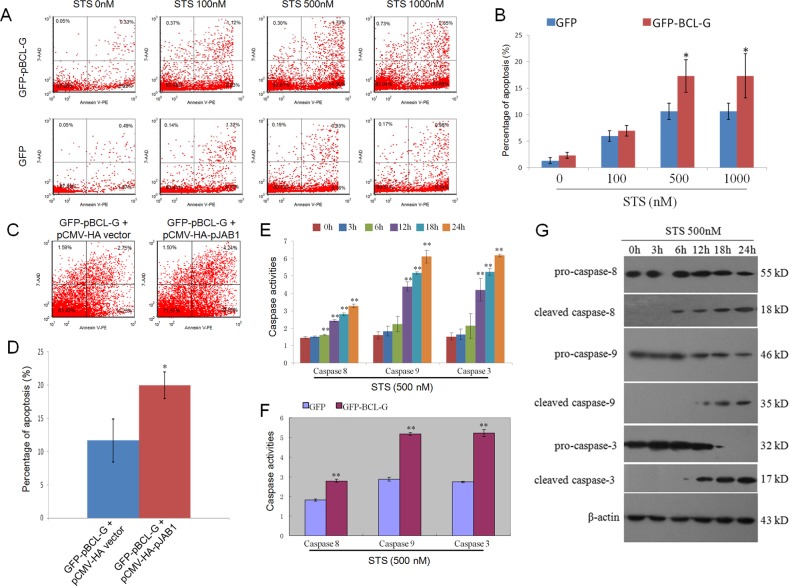
Quantification of apoptosis enhanced by interaction between pBCL-G and pJAB1 and detection of caspase activity **A.** Representative FCM diagrams of SUVEC cells stably expressing GFP-pBCL-G or GFP proteins treated with the corresponding STS doses for 18 hours. **B.** Quantification of annexin V-positive cells in Figure 7A. ^*^p < 0.05 *versus* the control group (Cells expressing GFP proteins). **C.** Representative FCM diagrams of SUVEC cells stably expressing GFP-pBCL-G proteins transfected with pCMV-HA-pJAB1 or pCMV-HA empty vector and treated with 500 nM STS doses for 18 hours. **D.** Quantification of annexin V-positive cells in Figure 7C. ^*^p < 0.05 *versus* the control group (Cells transfected with pCMV-HA empty vector). **E.** Detection of caspase activity. SUVEC cells stably expressing GFP-pBCL-G proteins were treated with 500 nM STS for indicated time. The activities of caspase-8, -9 and -3 in these cells were measured using the colorimetric assay kits. ^**^p < 0.01 *versus* the control group (0 hour). **F.** Effect of pBCL-G on caspase-8, -9 and -3 activities in STS-induced apoptosis. SUVEC cells expressing GFP-pBCL-G proteins and those expressing GFP proteins were both treated with 500 nM STS for 18 hours. The activities of caspase-8, -9 and -3 in these cells were measured using the colorimetric assay kits. ^**^p < 0.01 *versus* the control group (Cells expressing GFP proteins). **G.** Detection of the caspase activation by western blot. SUVEC cells stably expressing GFP-pBCL-G proteins were treated with 500 nM STS for indicated time. Whole cell lysates were analyzed by western blot to detect pro- and cleaved caspase-8, -9 and -3. The data shown are representative of three independent experiments. All experiments were performed in triplicate and data are expressed as means ± SD (*n* = 3). Error bars represent standard deviations of replicate data points.

In our previous study, pJAB1 was proved to enhance STS-induced apoptosis [11]. In this study, as mentioned above, pBCL-G was also proved to enhance STS-induced apoptosis. In consideration of the interaction of pBCL-G with pJAB1, we wonder whether STS-induced apoptosis can be enhanced when pBCL-G and pJAB1 are concurrently over-expressed in cells. To clarify this problem, SUVEC cells that could stably express GFP-pBCL-G protein were incubated with indicated doses of STS for 18 hours. Cells that could stably express GFP protein with the same treatment were used as a control. Then apoptosis was detected as mentioned above. As shown in Figure 7A and 7B, compared with cells that could stably express GFP protein, there was a significant increase in early apoptosis when cells that could stably express GFP-pBCL-G protein were treated with 500-1000 nM STS. The pCMV-HA-pJAB1 or pCMV-HA empty vector was then transfected into cells that could stably express GFP-pBCL-G protein, respectively. About 24 hours later, two groups of cells were both treated with 500 nM STS for 18 hours, followed by FCM to detect apoptosis. As shown in Figure 7C and 7D, compared with cells in which only pBCL-G protein was over-expressed, apoptosis was significantly enhanced when pBCL-G and pJAB1 proteins were both over-expressed in cells.

### Caspase-8, -9 and -3 are activated in apoptosis enhanced by pBCL-G

In this study, we investigated the mechanisms of apoptosis enhanced by pBCL-G. Caspases, especially caspase-8, -9 (the initiator caspases) and caspase-3 (the effector caspase), perform crucial function in various apoptotic signaling pathways. Therefore, we analyzed activities of these caspases in cells treated with STS. The results in Figure 7E showed that the activities of three kinds of caspases were all significantly increased in a time-dependent manner when SUVEC cells that could stably express GFP-pBCL-G protein were treated with 500 nM STS. Then, we explored the effect of pBCL-G on the activities of these caspases. The results in Figure 7F showed that three kinds of caspases in cells that could stably express GFP-pBCL-G protein were significantly activated compared with cells that could stably express GFP protein when both groups of cells were treated with 500 nM STS for 18 hours. Furtherly, western blot analysis was performed to confirm the activation of these caspases. The results in Figure 7G showed that the levels of cleaved caspase-8, -9 and -3 were increased when cells that could stably express GFP-pBCL-G protein were treated with 500 nM STS.

## DISCUSSION

In this study, we determined the expression pattern of the pBCL-G at transcript level, analyzed the influence of pivotal domains on the subcellular distribution of pBCL-G protein, verified the interaction of pBCL-G with pJAB1, and explored the impact of pBCL-G on STS-induced apoptosis.

The results of quantitative real-time PCR showed that pBCL-G mRNA was expressed in diverse tissues examined (Figure 1), which was consistent with the spatial expression of human BCL-G [3]. However, there is still some difference between the expression patterns of these two species. For example, the highest level of pBCL-G transcripts was detected in heart, followed by immune tissues such as abdominal lymph node, hilar lymph node, mesenteric lymph node, mandibular lymph node, spleen, and tonsil. In contrast, human BCL-G_S_ was expressed specifically in testis and the higher levels of human BCL-G_L_ transcripts was detected in lung, pancreas, prostate, and testis [3]. In previous studies, human BCL-G_L_ was reported to be correlated with multisystem autoimmune disease [30]. In addition, BCL-G can enhance apoptosis by interacting with JAB1 and MNSFβ [4, 31]. These reports suggest that BCL-G may act differently in different species and pBCL-G may correlate with functions of immune system.

The subcellular distribution of proteins that can be affected by some pivotal domains usually correlates with their functions [3]. As shown in our previous study, pBCL-G contains two typical domains of BCL-2 family, BH2 and BH3 domains [21]. To find out the pivotal domain of pBCL-G related with its subcellular distribution, two vectors that could express GFP-tagged pBCL-G proteins lacking the BH2 or BH3 domain respectively were generated and transfected into SUVEC cells. As shown in Figure 2B, the mutant pBCL-G protein lacking BH2 domain was distributed in a punctate cytosolic pattern whereas the mutant pBCL-G protein lacking BH3 domain and the complete pBCL-G protein were both distributed throughout cells. This result suggests that the BH2 domain is the pivotal domain that affects the subcellular localization of pBCL-G protein.

As mentioned previously, pBCL-G may interact with pJAB1 according to the sequence analysis. The co-immunoprecipitation assay was used to verify this interaction. As shown in Figure 4B, pBCL-G could interact with pJAB1. In human, the interaction of BCL-G_S_ with JAB1 depends on the N-terminal region of BCL-G_S_ [4]. Although the protein sequence of pJAB1 is identical to that of human JAB1, the similarity of protein sequences between pBCL-G and human BCL-G_L_ is only 71% [11, 21]. Therefore, the domains dependent on which pBCL-G interacts with pJAB1 need to be explored in the future studies.

In our previous studies, pJAB1 was distributed specifically in the cytoplasm while pBCL-G was distributed both in the cytoplasm and in the nucleus [11, 21]. Here, by confocal microscopy, the distribution of pBCL-G was affected when pJAB1 was over-expressed, which was confirmed by western blot analysis of cytoplasmic and nuclear fractions (Figure 5). These results also provided indirect evidence for the interaction of pBCL-G with pJAB1 to some extent.

In previous studies, STS-induced apoptosis could be enhanced by pJAB1 [11]. In consideration of the interaction of pJAB1 with pBCL-G, we explored the function of pBCL-G in STS-induced apoptosis. As shown in Figure 6B and 7B, STS-induced apoptosis was significantly enhanced by either transiently expressed pBCL-G or stably expressed pBCL-G. These results suggest that pBCL-G may take part in STS-induced apoptosis and characterization of the action mechanism may be helpful to build medical model for developing new cancer treatments.

Furtherly, we explored the probable mechanisms of apoptosis enhanced by pBCL-G. It is well known that caspases perform crucial function in apoptosis. Particularly, caspase-8 and -9 play pivotal roles in death receptor and mitochondrial apoptotic pathways, respectively. These two caspases can activate downstream caspase-3, which is the direct executor of apoptosis [32]. In our study, the activation of caspase-8, -9 and -3 was observed in STS-induced apoptosis (Figure 7E). Furthermore, the activation of these caspases was significantly increased by pBCL-G (Figure 7F). Therefore, our results indicate that STS-induced apoptosis can be enhanced by pBCL-G, which at least partially depends on the activation of caspase-8, -9 and -3.

Human JAB1 could interact with BCL-G_S_, enhancing mitochondrial apoptosis [4]. pBCL-G shared 71% identity with human BCL-G_L_ [21]. Here, our results demonstrated that pBCL-G and pJAB1 synergistically promoted STS-induced apoptosis (Figure 7G). Therefore, human JAB1 may also interact with BCL-G_L_. Taken together, the mechanism of apoptosis enhanced by JAB1 need to be studied further.

In conclusion, we clarified the expression pattern of pBCL-G mRNA, determined that the subcellular distribution of pBCL-G could be affected by its BH2 domain, proved that pBCL-G could interact with pJAB1, and that this interaction may significantly enhance STS-induced apoptosis.

It is evident that more work need to be done to unveil the whole mechanisms of apoptosis promoted by pBCL-G. Some critical questions should be answered in the further study. For instance, why were caspase-8, -9 and -3 activated in apoptosis enhanced by pBCL-G? How does pBCL-G take part in STS-induced apoptosis? Work ongoing in our laboratory is trying to address these issues. Our study provides a potential molecular model to explore the role of BCL-G in the correlated diseases.

## MATERIALS AND METHODS

### Ethics statement

Our study had been approved by Animal Care and Use Committee of Shaanxi Province, China. All animal procedures were performed according to guidelines developed by the China Council on Animal Care and protocol approved by Animal Care and Use Committee of Shaanxi Province, China.

### Animals, cells and genes

The 1-month-old large white pigs were housed and had been approved by the scientific ethical committee of the Northwest A&F University before it was used in this study. The SUVEC cells were cultured as previously described [33]. pBCL-G and pJAB1 genes were respectively cloned from porcine spleen and heart by reverse transcription-PCR and stored in the Laboratory of Virology, Immunology and Bioinformatics, College of Veterinary Medicine, Northwest A&F University [11, 21].

### Reagents

Dulbecco's modified eagle medium and fetal bovine serum were obtained from Hyclone (Hyclone, Beijing, China). Staurosporine was obtained from Sigma Aldrich (Sigma Aldrich, Saint Louis, USA). Mouse monoclonal antibodies against GFP-tag, and Beta-actin (β-actin) were purchased from Santa Cruz Biotechnology, Inc. (Santa Cruz, CA, USA). Rabbit polyclonal antibodies against caspase-8 and caspase-3 were purchased from Abcam Inc. (Boston, MA, USA). Rabbit polyclonal antibody against caspase-9 was purchased from Biovision Inc. (Milpitas, CA, USA). Horseradish peroxidase (HRP)-conjugated rabbit anti-mouse secondary antibody and goat anti-rabbit secondary antibody were purchased from Sigma Aldrich (Sigma Aldrich, Saint Louis, USA).

### Plasmid construction

The cDNA containing the open reading frame (ORF) of pBCL-G without additional flanking sequences was generated by PCR with the following primer pair: forward, 5′-AAGTCGACCATGTGCACCACCAGC-3′ and reverse, 5′-CCGGTACCTCAGTCTACTTCTTCATGGG-3′. The PCR products were digested with restriction endonucleases of *Sal* I and *Kpn* I and subcloned into the corresponding sites of pCMV-myc vector (BD Biosciences Clontech, CA, USA). The recombinant vector was named pCMV-myc-pBCL-G. The cDNA containing the ORF of pJAB1 without additional flanking sequences was generated by PCR with the following primer pair: forward, 5′-TAGAATTCATGGCGGCTTCTGGGAG-3′ and reverse, 5′-GCGGTACCTTAAGAGATGTTAATTTGAT-3′. The PCR products were digested with restriction endonucleases of *EcoR* I and *Kpn* I and subcloned into the corresponding sites of pCMV-HA vector (BD Biosciences Clontech, CA, USA). The recombinant vector was named pCMV-HA-pJAB1.

### Real-time PCR analysis of spatial expression patterns

To analyze tissue distribution of pBCL-G in eleven tissues (heart, liver, spleen, lung, kidney, thymus, tonsil, hilar lymph nodes, mandibular lymph nodes, mesenteric lymph nodes, Abdominal lymph nodes) obtained from three 1-month-old, large white pigs, quantitative real-time RT-PCR was carried out as previously described [11]. The primers used are described as following: pBCL-G forward, 5′-GCTCTGCTGTCTTCTCACCAAA-3′ and reverse, 5′- ATTTTCCTCCTTCTCTGCTACTCC-3′; β-actin forward, 5′- CAAGGACCTCTACGCCAACAC-3′ and reverse, 5′- AGGATGGAGCCGCCGATC-3′.

### Effect of pivotal domains on the subcellular distribution of the pBCL-G protein

To study the impact of pivotal domains on the subcellular distribution, the pBCL-G cDNA was subcloned into the pEGFP-C1 vector to generate the recombinant expressing vector pEGFP-pBCL-G as previously described [21]. Then, two mutants of pBCL-G lacking the BH3 or BH2 domain were created by an overlap PCR method respectively. The resulting PCR product was also subcloned into the pEGFP-C1 vector to generate the recombinant expressing vector pEGFP-pBCL-G-BH3-deficient or pEGFP-pBCL-G-BH2-deficient. The vector was sequenced for accuracy. The primers used are described as following: primer1, 5′-AAGGTACCATGTGCACCACCAGC-3′; primer2, 5′-GCGGATCCTCAGTCTACTTCTTCATGGGA-3′; primer3, 5′- GTCATCAAAATGCTCTTCGCACTTGGAGCTCT-3′; and primer4, 5′-AGGGATCCTCACGAGAAGTTCTCCTTCAG-3′. SUVEC cells were respectively transfected with three kinds of vectors and the expression of three kinds of GFP fusion proteins was detected by western blot analysis. The subcellular localization of indicated GFP fusion proteins was detected with A1R MP Confocal Microscope (Nikon Instruments, Tokyo, Japan) as previously described [11].

### Establishment of cell line stably expressing GFP-pBCL-G protein

SUVEC cells were cultured in 6-well plates for 24 hours as previously described [33], and then in medium without serum for 1 hour. The cells were transiently transfected with 4 μg of the pEGFP-pBCL-G or pEGFP-C1 vectors, combined with Lipofectamine2000 (Invitrogen, Carlsbad, CA, USA) according to the manufacturer's instructions. 24 hours after transfection, complete DMEM was supplemented with G418 (800 μg/mL) to select the cell lines stably expressing GFP-pBCL-G or GFP proteins. The selected cell lines were analyzed using inverted fluorescent microscopy and confirmed by western blot analysis.

### Indirect immuno-fluorescence assay

To detect the expression of myc-pBCL-G and HA-pJAB1, SUVEC cells were seeded in 35-mm petri dishes (MatTek, MA, USA) and were co-transfected with pCMV-myc-pBCL-G and pCMV-HA-pJAB1 vectors. About 24 hours after transfection, cells were fixed with 4 % paraformaldehyde for 30 minutes at room temperature and permeabilized with 0.2 % Triton X-100/PBS for 5 minutes. Then, cells were incubated with mouse anti-myc tag (Sigma Aldrich, Saint Louis, USA) or anti-HA tag (Sigma Aldrich, Saint Louis, USA) monoclonal antibody (1:1000 dilution) for 1 hour at room temperature, followed by incubation with FITC-conjugated goat anti-mouse IgG secondary antibody (Sigma Aldrich, Saint Louis, USA, 1:100 dilution) for 1 hour at room temperature. After being rinsed with PBS, cells were observed using an inverted fluorescence microscope.

### Co-immunoprecipitation

Co-immunoprecipitation was performed with a Pierce^®^ Mammalian c-Myc Tag IP/Co-IP Kit as per the manufacturer's instructions. Firstly, SUVEC cells were transfected with a mixture of pCMV-myc-pBCL-G and pCMV-HA-pJAB1 vectors while cells were transfected with a mixture of pCMV-myc-pBCL-G and pCMV-HA empty vector or with a mixture of pCMV-HA-pJAB1 and pCMV-myc empty vector as control groups. About 36 hours after transfection, protein samples were extracted from indicated cells using the M-PER^®^ Mammalian Protein Extraction Reagent. Samples were then incubated overnight at 4°C with 10 μL of anti-c-Myc agarose slurry. The agarose slurry was washed with a wash solution of Tris Buffered Saline plus 0.05% Tween-20 for 3 times and immunoprecipitates were released from the agarose slurry by boiling in 25 μL non-reducing sample buffer for 5 minutes. Western blot was carried out with anti-HA tag or anti-myc tag antibody.

### Analysis of apoptosis by flow cytometry

To study whether pBCL-G gene has any function related to STS-induced apoptosis, we detected the apoptosis of SUVEC cells transiently or stably expressing GFP-pBCL-G induced by STS, respectively. The FCM was performed as described in previous studies with minor modifications [11]. The FCM system CyFlow Cube6 (Partec, Amtsgericht Dresden, Germany) and annexin V-PE/7-AAD kit (BD Bioscience, Rockville, MD, USA) were used according to the manufacturer's instructions. SUVEC cells transiently or stably expressing GFP-pBCL-G were plated in six-well plates at a concentration of 2.5×10^5^ cells per well (2 mL/well) and cultured as previously described, respectively. When about 80% confluence was observed, the medium was replaced with fresh challenge media supplemented with different doses of STS. Cells were harvested at 18 hours after treatment. After being washed with D-Hanks, the cells were detached with trypsin and 5×10^5^ cells of each group were resuspended in binding buffer and incubated with annexinV-PE/7-AAD for 15 minutes at 25°C. Dead cells and debris were excluded by selective gating based on electronic cell volume. About twenty thousand events were collected from each sample. By using dual-parameter staining, early apoptosis was quantified, using the CyView8.5 software (Partec, Amtsgericht Dresden, Germany). Apoptosis in cells transiently or stably expressing GFP with the same treatment was respectively detected as a control. In preliminary experiments, unstained medium-treated cells were used to define living cells (lower left quadrant). Cells treated with H_2_O_2_ as positive control were singly stained with annexin V-PE or 7-AAD to define respectively the lower right and upper left quadrants.

### Caspase activity assay

Activities of caspase-8, -9 and -3 were measured by colorimetric assay kits (Biovision, Inc., Mountain View, CA, USA) according to the manufacturer's instructions as described in previous studies [11]. Briefly, cells were collected and lysed on ice and protein concentration was measured using BCA Protein Assay Reagent (Pierce, Rockford, IL, USA), then 200 μg of protein was incubated with each caspase substrate at 37°C in a microplate for 4 hours. The resultant color was read at 405 nm in Thermo Scientific Multiskan FC microplate photometer (Thermo Fisher Scientific Inc., Vantaa, Finland). The activation of caspases was further confirmed by western blot analysis.

### Western blot

Protein samples were extracted from indicated cells using lysis buffer (50 mM Tris-HCl, 5 mM EDTA, 150 mM NaCl, 0.1% NP-40, 0.5% deoxycholic acid, 1 mM sodium orthovanadate, 100 μg/mL PMSF, and protease inhibitors). For western blot analysis of nuclear and cytoplasmic fractions, cell nuclear and cytoplasmic fractions were prepared using a nuclear/cytosol fractionation kit (Biovision, Mountain View, CA) as per the manufacturer's instructions. The protein concentration was determined using the BCA Protein Assay Kit (Pierce, Rockford, IL, USA). Equivalent amounts of protein samples were uploaded and separated by SDS-PAGE and then transferred to a polyvinylidene fluoride membrane (Millipore, Billerica, MA, USA). The membrane was blocked with blocking buffer (5 % skimmed milk in Tris-HCl buffered saline) and then incubated with indicated primary antibodies over night at 4°C, followed by HRP-conjugated secondary antibodies at room temperature for 1 hour. The protein bands were visualized by Luminata Classico Western HRP Substrate as per the manufacturer's instructions (Millipore, Billerica, MA, USA).

### Statistical analysis

Calculation of means and standard deviations (SD) and statistical analysis were performed with SPSS 17.0. Differences between test and control groups were assessed using independent Samples Test, with p-value below 0.05 considered statistically significant.
